# A simplified diagnostic work-up for the detection of gestational diabetes mellitus in low resources settings: achievements and challenges

**DOI:** 10.1007/s00404-020-05708-x

**Published:** 2020-07-30

**Authors:** Giovanni Putoto, Edgardo Somigliana, Federico Olivo, Simona Ponte, Michael Momoh Koroma, Federica Citterio, Michele Orsi, Enzo Pisani, Marica Pilon, Fabio Manenti, Giulia Segafredo

**Affiliations:** 1 Doctors with Africa CUAMM, Padua, Italy; 2 Università degli Studi di Milano, Via M. Fanti, 6, 20122 Milan, Italy; 3 Fondazione IRCCS Ca’ Granda Ospedale Maggiore Policlinico, Milan, Italy; 4 Doctors with Africa CUAMM, Freetown, Sierra Leone; 5 Princess Christian Maternity Hospital, Freetown, Sierra Leone

**Keywords:** Gestational diabetes mellitus, Screening, Low-resource setting, Glucose

## Abstract

**Purpose:**

Modern strategies for the screening and diagnosis of Gestational Diabetes Mellitus (GDM) rely on universal Oral Glucose Tolerance Test (OGTT). However, they are unsustainable in low-income countries. In this study, we aimed at assessing the feasibility of a simplified diagnostic policy.

**Methods:**

The study took place in an urban referral hospital in Freetown, Sierra Leone. During an 11-month period, pregnant women were offered capillary blood test for glucose assessment. They could be screened at any time during pregnancy. GDM was diagnosed if fasting glucose was ≥ 92 mg/dl or if the OGTT was positive. The latter was prescribed only to women presenting after 24 weeks’ gestation with at least one risk factor for GDM and fasting capillary glucose between 85 and 91 mg/dl. A definitive diagnosis required confirmation to this aim, women with values above the thresholds were invited to refer the next working day for repeating the test after fasting overnight.

**Results:**

Overall, 7827 women were referred for screening, of whom 6872 (87%) underwent at least one capillary glucose assessment. However, 895 of those who had a positive test did not return for confirmation. Overall, a definite assessment could be done in 5799 subjects corresponding to 76% (95% CI 75–77%) of those eligible. GDM was diagnosed in 128 women (1.9%, 95% CI 1.6–2.2%). Based on an expected confirmation rate of 22% (calculated from those who referred for confirmation) in the 895 women who did not come back, one could infer that GDM would have been diagnosed in additional 197 women, raising the prevalence to 4.7% (95% CI 4.2–5.3%).

**Conclusion:**

Three quarters of subjects could be assessed with our approach. Data also suggest that GDM is not rare even if identification of affected cases remains challenging.

## Introduction

There has been a remarkable progress in health status in low-resource countries over the past two decades. However, additional important efforts are required to improve health and reduce mortality, particularly maternal and newborn mortality [1].

Gestational Diabetes Mellitus (GDM) in low-resource settings is neglected and has been poorly studied [2, 3]. However, improving the management of this condition may represent a valuable action to further tackle maternal and neonatal mortality and morbidity. Indeed, GDM is associated with obstetrical and neonatal complications that may expose women and their children to short- and long-term risks [4–7]. The hyperinsulinism can cause excessive foetal growth with metabolic consequences that can persist in childhood and adult life [5]. In the short term, the overgrowth of the foetus can cause complications at birth (traumatic injury, shoulder dystocia, obstructed labour and caesarean section). In addition, GDM increases the risk of preeclampsia and puerperal sepsis [4, 6, 8]. Of relevance is that pivotal randomized-controlled trials (RCTs) consistently showed that a prompt identification and treatment of GDM can effectively prevent these complications [9, 10]. On these bases, the main international scientific societies advocate universal screening of GDM with Oral Glucose Tolerance Test (OGTT) [11]. Specifically, based on the International Association of Diabetes in Pregnancy Study Groups (IADPSG) criteria, GDM is diagnosed if at least one of the three peripheral blood assessments of the 75 g OGTT overcomes the thresholds (baseline fasting ≥ 92 mg/dl, 1-h ≥ 180 mg/dl and 2-h ≥ 153 mg/dl) [12].

On the other hand, inferences of evidence obtained in western world to low-resource countries are challenging. Gold standards of management are actually unsustainable in these settings. Main obstacles include the lack of infrastructures, the scant awareness of the condition and of the possible preventive interventions, the low compliance and adherence of patients and the lack of financial resources to cover the costs [13, 14]. In addition, the clinical relevance of GDM in low-income countries remains uncertain. However, one may hypothesize that GDM-related complications could be more devastating because access to caesarean section and intense neonatal and adult care is hindered or significantly delayed. In addition, the strict monitoring of foetal conditions that is generally advocated for GDM women to prevent overgrowth or metabolic consequences is more complicated.

In this cross-sectional study, we aimed at assessing the feasibility of a simplified policy for the detection of GDM in Sierra Leone. The country is an underprivileged area of Sub-Saharan Africa ranking 184 out of 189 on the Human Development Index [15], with a under-5 mortality rate of 110 deaths per 1000 live births and a maternal mortality rate of 1,360 per 100,000 live births [16, 17]. More specifically, consecutive women referring for antenatal visits in a referral urban hospital were offered to be screened with mere capillary testing, regardless of the gestational age. OGTT was prescribed after 24 weeks’ gestation exclusively to women with risk factors and borderline glucose values at baseline evaluation. The primary aim was determining the rate of women that could be assessed with this policy.

## Methods

The study took origin from a comprehensive 2 years lasting project specifically dedicated to improving the management of GDM in the Republic of Sierra Leone. The project was supported by the World Diabetes Foundation (WDF) and implemented by the Italian non-governmental organization (NGO) Doctors with Africa CUAMM. The present cross-sectional study reports on part of the program, more specifically on the screening of GDM performed at the Princess Christian Maternity Hospital, a public referral hospital located in Freetown (the capital of the Republicof Sierra Leone). The study was approved by the local Institutional Review Board. An informed consensus was not requested because the study was planned after the implementation of the project and data were collected retrospectively.

The Princess Christian Maternity Hospital receives referrals for obstetrics complications from a catchment area of 1,606,145 inhabitants, corresponding to an annual number of expected pregnancies of 64,246. In 2018, the hospital ensured 22,542 antenatal visits, of whom 9440 were first visits. The total number of deliveries in the year was 7367, corresponding to 19% of all institutional deliveries in the area (data inferred from Sierra Leone 2015 Population and Housing Census, 2015) [18].

The main idea of the project was to determine the effectiveness and validity of GDM screening and diagnosis in low-resource settings, taking into utmost consideration the issues of sustainability and feasibility. The main views included the potential clinical relevance of GDM in low-resource settings [2] as well as the idea that glucose intolerance in pregnancy is not a “yes or no” condition but, instead, a situation characterized by a linear gradient between the grade of glucose intolerance and the frequency and severity of GDM-related obstetrics complications [5]. In other words, we did not aim at blindly transferring the western world modality of GDM detection, but, conversely, we aimed at implementing a sustainable screening that could detect the most worrisome cases. The project initiated in April 2017 and lasted 2 years. Data reported herein were collected starting some months after the implementation to allow the system to properly run. Specifically, data were retrieved from March 3, 2018 to January 30, 2019 with a 2-week interruption at the end of March because of the accidental loss of the dedicated registers.

**Fig. 1:**
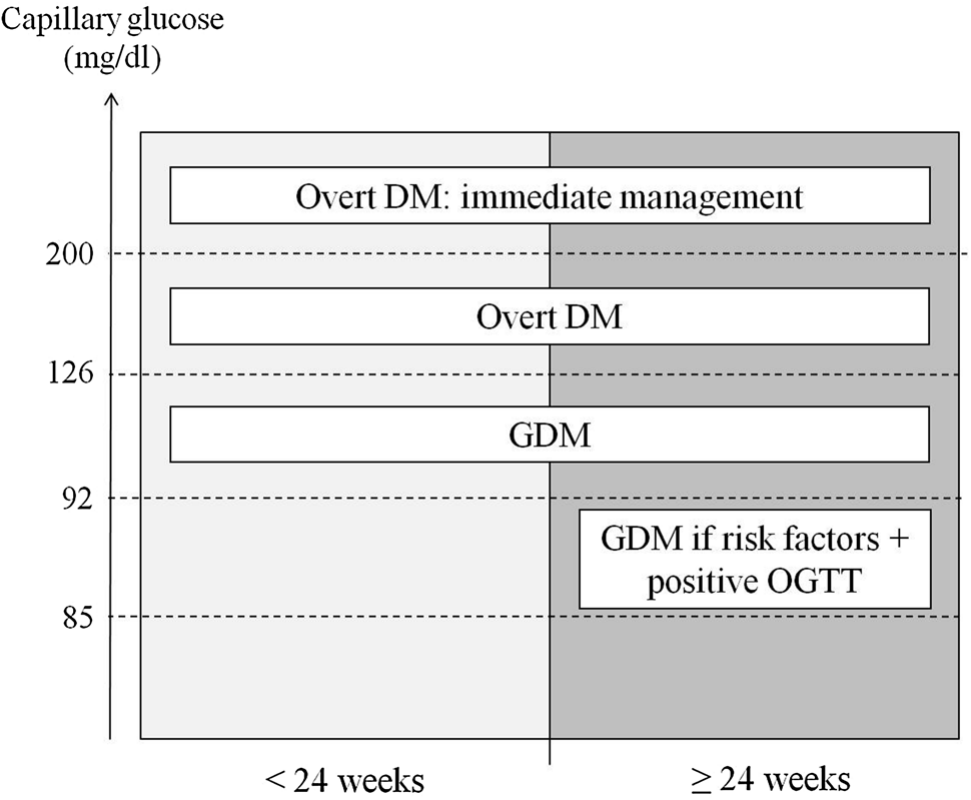
Criteria for GDM diagnosis. Women were tested before and after 24 weeks’ gestation. Diagnosis of GDM was done if fasting capillary glucose levels were ≥ 92 mg/dl (overt DM was diagnosed for levels ≥ 126 mg/dl). Women with levels exceeding 200 mg/dl were promptly referred for immediate clinical management. To rule out false positive results, women with positive findings were invited to refer the following working day after fasting overnight for confirmation (“come back tomorrow”). In addition, after 24 weeks’ gestation, the subgroup of women presenting with risk factors for GDM and borderline fasting capillary glucose levels (85–91 mg/dl) underwent a 75 gOGTT. Criteria for diagnosis with OGTT were the detection of at least one value above the thresholds of 92, 180 and 153 mg/dl at baseline, and one and two hours after the glucose load, respectively. *GDM* gestational diabetes mellitus, *DM* diabetes mellitus, *OGTT* oral glucose tolerance test

A team of expatriated gynaecologists and midwifes with expertise in both western world and low-income settings implemented the project and performed the teaching and tutoring. Since the beginning of the project, a service specifically dedicated to GDM management and run by two trained nurses was implemented at the hospital. The criteria used for the diagnosis of GDM are illustrated in Fig. 1. Specifically, all women referring to the antenatal ward for obstetrical care were offered to be screened twice, one before and one after 24 weeks’ gestation. No further specific restrictions for gestational age were given. The cut-off of 24 weeks’ gestation was chosen because there is a general consensus that screening for GDM should be done after this gestational age [8–10]. However, given the elevated proportion of women referring for antenatal care only once during pregnancy in our setting, we could not restrict the time for screening only to the second part of pregnancy. We, thus, deemed important to perform the GDM assessment regardless of gestational age. Women accepting to perform the screening were referred to the GDM room to be managed by the dedicated nurses. The presence of risk factors was actively and systematically investigated. They included a first-degree relative with diabetes mellitus, BMI > 30 kg/m^2^, age > 35 years, history of GDM, repeated miscarriages (≥ 3), previous stillbirth and previous macrosomia (neonatal weight > 4 kg). In women reporting fasting, a capillary blood sample was obtained and immediately tested for glucose concentration using a glucometer. Women denying fasting and those with values exceeding the stated limits (Fig. 1) were invited to refer the next working day for re-check capillary glucose concentration (“come back tomorrow”) underlying the crucial importance of overnight fasting. This strategy was decided soon after the initiation of the project when it became evident that information regarding fasting was poorly reliable. It was, thus, decided that only women whose values exceeded the stated thresholds at the second assessment were diagnosed with GDM and continued in the diagnostic and therapeutic work-up. The unique exception to the “come back tomorrow” rule was the finding of capillary glucose exceeding 200 mg/dl at the first assessment; these women were straight referred to medical doctors for prompt management. In addition, women at gestational ages > 24 weeks’ gestation with at least one risk factor for GDM and confirmed fasting capillary glucose between 85 and 91 mg/dl underwent a 75 g OGTT. They were diagnosed with GDM if at least one of the three assessments overcame the thresholds of 92 (basal), 180 (1 h) and 153 (2 h) mg/dl. Testing for OGTT was also done using capillary blood assessments. The idea to limit the OGTT exclusively to this subgroup of women rather than to the whole population was taken to avoid overburdening the antenatal care service.

In case of GDM diagnosis, women were invited to refer weekly. At first assessment, they were invited to increase physical activity if sedentary and to restrict food assumption if overweight. They also received a dietary consultation to improve their diet and were given a specific informative flyer. At each weekly visit, women presented after overnight fasting and capillary glucose were assessed before eating and 2 h later. The values obtained were used to tailor treatment and to decide on the need for medical therapies (metformin, glyburide or insulin).

Gestational age was calculated based on last menstrual period. In case of major discrepancy between gestational age and uterus measurement, an ultrasound examination was performed and, if indicated, gestational age was adjusted. Women referring to the antenatal care service did not routinely undergo ultrasound scanning. However, this examination was done in case of suspected pregnancy complications. Conversely, women who were diagnosed with GDM were systematically scanned at the time of GDM diagnosis and subsequently at every referral to monitor foetal growth and well-being. Elective caesarean section was planned in case of estimated foetal weight above 4500 g. Foetal growth exceeding the 90th percentile was an indication for induction of labour if the woman had reached at least 38 weeks’ gestation.

Capillary glucose assessments were generally done using the glucometer On Call Plus II^®^ (San Diego, CA, US). The HemoCue® glucose 201 RT (Angelholm, Sweden) was used for the OGTT and for GDM monitoring.

All diagnostic steps were for free. They include the capillary assessments, the consultations and the ultrasound scans if needed. Conversely, medical treatments were not supported by the project (subjects had to pay for the drugs). Women requested to come back the following working day after a fasting overnight were refunded of the costs of the journey.

Follow-up of affected women was done by consulting patients’ charts at the hospital.

Data analysis was done using the Statistical Package for Social Sciences software (SPSS version 25.0, IL, USA). The recruited sample size was deemed sufficient to provide an estimate of the frequency of tested women and GDM diagnoses with a 95% Confidence Interval (CI) of less than 2%. The 95% CIs of proportions were calculated using a binomial distribution model. The Kaplan–Meier method was used to obtain the survival curve on the cumulative risk of GDM with gestational age. The frequency of risk factors between affected and unaffected women was compared using the Fisher’s exact test. For this analysis, missing data were equated to absence of risk factor. The database is available on request. The described intervention ended in August 2019 and further evaluations are not foreseen.

## Results

**Fig. 2:**
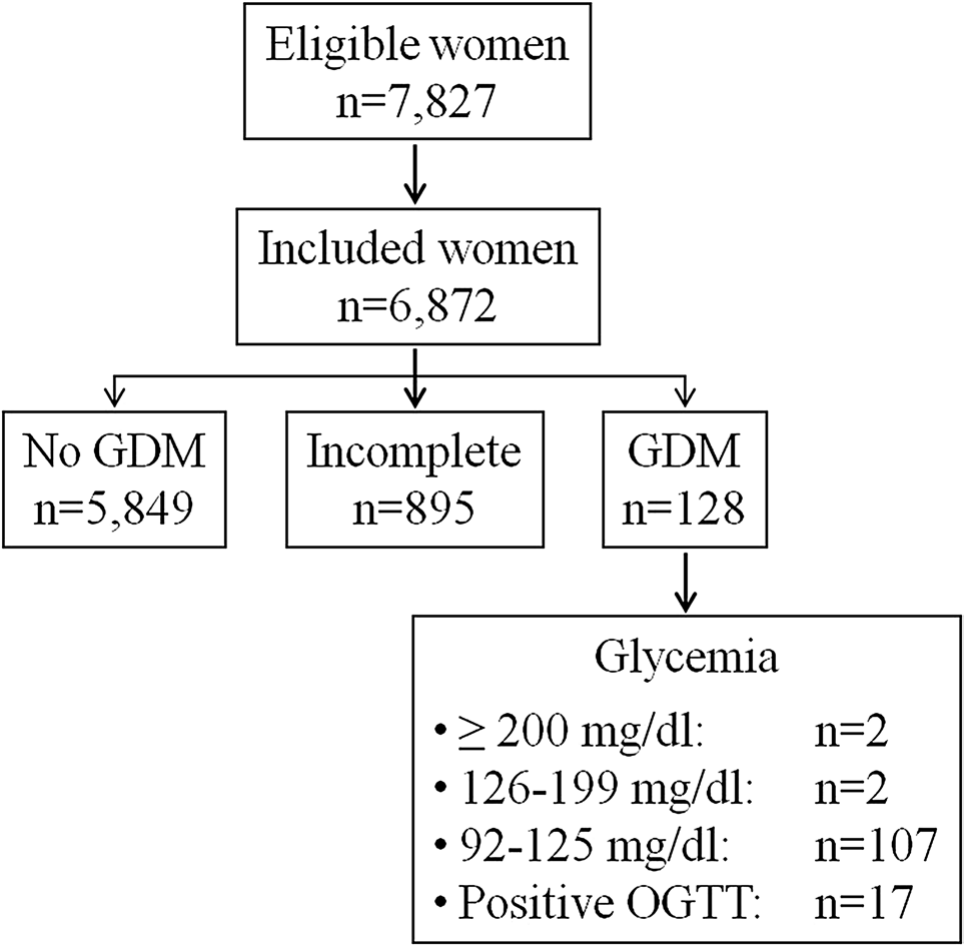
Flow diagram of the study. Women were classified as “incomplete” when they were requested to come back the following day for re-assessment because of fasting capillary glucose levels exceeding 92 mg/dl but they never came back

The flow diagram of the studied women is shown in Fig. 2. Overall, during the study period, 7827 women were referred for screening. In 955 of them, capillary testing could not be done because they reported to have just eaten and, despite being invited to refer again the following working day, they never came back. They were judged non-informative. Overall, data were available for 6872 women, corresponding to 87% of those eligible. Nulliparity was reported in 2689 subjects (39%); 2270 (33%) had one previous delivery, 1084 (16%) had two previous deliveries and 790 (12%) had three or more previous deliveries.

One-thousand and nine-hundred sixty women (29%) were screened exclusively before 24 weeks’ gestation, 3744 (54%) exclusively after 24 weeks’ gestation and the remaining 1168 (17%) underwent both assessments. Overall, 4912 women were screened in the second part of pregnancy. OGTT was required in 32 women.

GDM was diagnosed in 128 women while the screening resulted negative in 5849 women. The remaining 895 women with initial positive capillary testing requiring confirmation did not perform it; 864 of them did not return despite the recommendation to come back the following day without providing explanations (drop-outs), while in 31 cases, this was due to the violation of the clinical protocol, i.e., they came back but did not undergo the test. Overall, definite information on GDM was, thus, available in 5977 women (128 + 5,849), corresponding to 87% (95% CI 86–88%) of those included and 76% (95% CI 75–77%) of those eligible. The diagnosis was exclusively based on fasting testing in 111 women (in four cases values were above 126 mg/dl) corresponding to 87% of the identified cases, whereas OGTT was required in the remaining 17 (13%). Forty seven out of 128 cases (37%) were diagnosed before 24 weeks’ gestation; the remaining 81 (63%) were identified after 24 weeks’ gestation.

Risk factors for GDM were more common in affected cases with the exception of a history of GDM and recurrent miscarriages (Table 1). Seventy six affected women (59%) had at least one risk factor compared to 1,404 controls (21%, *p* < 0.001).

**Table 1:** Risk factors for GDM in women with and without a diagnosis of GDM

Characteristics	GDM	Controls	*p*
Totale number	128	6744	
Age ≥ 35 years	19 (15%)	548 (8%)	0.02
BMI > 30 kg/m^2^	28 (22%)	986 (15%)	0.04
Previous GDM	3 (2%)	50 (1%)	0.15
First degree relatives with DM	10 (8%)	55 (1%)	< 0.001
Recurrent miscarriages	3 (2%)	48 (1%)	0.07
Previous stillbirths	10 (8%)	66 (1%)	< 0.001
Previous macrosoma	22 (17%)	70 (1%)	< 0.001

Missing values were considered as negative

*GDM* gestational diabetes mellitus, *DM* diabetes mellitus, *BMI* body mass index

To assess the importance of confirmation and its possible confounding effect on the estimation of the prevalence of GDM, we did a subgroup analyses on all women who attended for confirmation (*n* = 577). The frequency of women who were diagnosed GDM (*n* = 128) was 22%.

**Fig. 3:**
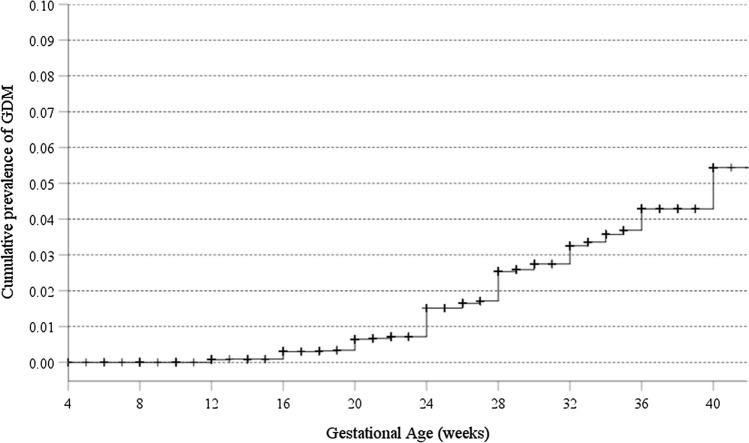
Survival curve on the cumulative risk of GDM according to gestational age. The Kaplan-Meyer method was used to draw the curve. The prevalence of GDM at 24, 32 and 40 weeks’ gestation was 1.5%, 3.3% and 5.4%, respectively

Overall, the prevalence of GDM in our cohort was 1.9% (95% CI 1.6–2.2%) (128/6,872). Figure 3 illustrates the cumulative rate of GDM with gestational age. Based on an expected confirmation rate of 22% in the 895 women who did not come back, one could infer that GDM would have been diagnosed in additional 197 women. The inferred prevalence of GDM in our cohort would raise to 4.7% (95% CI 4.2–5.3%) (128 + 197/6872).

Among women with GDM, the rate of referral for subsequent monitoring was 77%, 72%, 65%, 55% and 44% at 1st, 2nd, 3rd, 4th and 5th weeks, respectively. Insulin was immediately prescribed in the 4 women with overt Diabetes Mellitus. Twenty additional women were prescribed metformin or glyburide because diet was insufficient. None of the women who referred regularly were given insulin. Data on pregnancy outcome were available in 50 out of the 128 GDM cases (39%). One maternal death occurred in a woman because of eclampsia. Six stillbirths were documented (including the one associated to the maternal death). Among women delivering viable foetuses (*n* = 44), the rate of macrosomia (foetal weight > 4 kg) was 32% (*n* = 14, of whom seven underwent caesarean section). The rate of caesarean section was 18% (*n* = 8).

## Discussion

GDM is not rare in low-income countries but identification of affected cases is challenging. Our protocol was shown to be acceptable by the local population since the vast majority of women accepted the screening. Overall, definite data on GDM were available in three quarters of eligible subjects (76%) and six of seven included women (87%). However, we detected less than half of the potentially affected cases, i.e., 1.9% rather than a projected 4.7% that would have occurred if adherence to our confirmation policy was optimal. In addition, given the increase in prevalence with gestational age [19], the number of detected cases could have been higher if women referred more frequently in the second part of pregnancy. Indeed, the cumulative prevalence of GDM in our cohort overcomes 5% at the end of pregnancy. In fact, 29% of women in our cohort could be tested only in the first part of pregnancy. To note, based on a theoretical simulation that we run hypothesizing to overcome these two pitfalls (i.e., all women returned for confirmation and all were tested in the second part of pregnancy), the cumulative prevalence of GDM would reach 12% at the end of pregnancy (data not shown).

Even if there is currently a general consensus on the criteria to be applied for GDM diagnosis [12], their use in low-resource settings remain challenging and mostly inapplicable. The time required to perform an OGTT, the need for a specific gestational age for testing (24–28 weeks’ gestation) and the need for blood rather than capillary assessments hamper its universal application. In a recent systematic review, Behboudi-Gendevani et al. identified seven main strategies for GDM screening [3]. The most appealing for low-resource countries is the 75 g OGTT with a single assessment at 2 h and a threshold for diagnosis set at 140 mg/dl. The pros include the possibility to perform the test also in non-fasting women and the need for a single rather than multiple assessments. The cons are the needs to prolong antenatal visits for at least 2 h and the necessity for a blood rather than a capillary assessment. This latter aspect actually hinders its use in peripheral basic care facilities that rarely have the possibility to perform blood tests. As a matter of fact, albeit attracting, this option appears inappropriate for universal application. If validated in other contexts, a screening approach exclusively using capillary assessments like the one proposed in the present study could be more sustainable.

In general, our study shows that GDM is not rare and screening may be worthwhile. To the best of our knowledge, the present study represents the first population-based evidence on the prevalence of GDM in the Republic of Sierra Leone and one of the few in sub-Saharan countries [2, 3]. To note, previously reported prevalence in this geographical area varied between 0 and 14% [2, 3], and thus in line with our findings. However, reliable epidemiological comparisons with other countries cannot be done. The use of peculiar and arbitrarily set criteria of diagnosis rather than the referral 75 g OGTT at 24–28 weeks hampers meaningful comparisons. On the other hand, it should be underlined that a population-based study applying the standard criteria of diagnosis in sub-Saharan countries is currently utopian. The few available evidence adopting these criteria in low-resource settings was obtained in experimental contexts [20, 21]. In addition, the authors had to face a consistent proportion of drop-outs (close to 50%), a situation that exposes the results to potential confounders [21]. In this regard, our study better reflects real-life situations. We actually opted to favour sustainability rather than methodology. However, our arbitrary criterion for definition of GDM is a weakness of the study and validation is warranted. Of particular concern is the reliability of capillary compared to plasma assessments of peripheral glucose. This aspect remains debated in general and the use for OGTT is not validated [22–24].

The Hyperglycaemia and Adverse Pregnancy Outcome (HAPO) study showed that there is a linear relation between hyperglycaemia in pregnancy and obstetrics complications [8]. The International Association of Diabetes in Pregnancy Study Groups (IADPSG) criteria for GDM diagnosis were set based on the wise but arguable statement that an increase in risk of complication of 1.75 would be relevant [12]. Therefore, so far, any thresholds for diagnosis could be viewed as arbitrary. In addition, one has to underline that, in the HAPO study, the increase in risk with the increase in hyperglycaemia was documented for all the three assessments of the OGTT (baseline, 1 h and 2 h). Using three measurements rather than only the basal one increased the sensitivity but not the specificity. In fact, 55% of cases were captured exclusively with the basal assessment (and this rate overcame 70% in some of the participating centers) [8]. On this basis, one may wonder whether the OGTT could be completely omitted in the diagnostic work-up in low-resource settings (at least in the initial phase of implementation), in particular if one aims at universal coverage and, thus, needs to include Peripheral Health Centers. Performing OGTT also requires fasting and makes the screening more complex and expensive. In addition, our data show that confirmation could be more fruitful in the identification of additional cases than OGTT. To note, this may also be due to our protocol that gave the indication to OGTT only in women with risk factors, a situation that was not very common (Table 1). In this regard, it has also to be pointed out that previous GDM was inevitably rare given that a policy of systematic screening was not in place. In addition, the relatively high proportion of nulliparous (39%) hinders the relevance of risk factors, such as previous stillbirths, previous GDM and previous macrosoma. Of interest here is also that differences in the frequency of risk factors markedly differ between affected and unaffected cases only for rare exposures (previous GDM, previous stillbirths and previous macrosoma) (Table 1), thus confirming the scant validity of a strategy based on risk factors for the identification of GDM cases. In fact, risk factors were present in only 3 out of 5 cases.

Overall, we believe that the main point of discussion is not whether or not our strategy identified all GDM cases (it did not) but, conversely, whether we captured those at highest risk of complications. We are confident that this might be the case, but we have to recognize that this opinion is speculative and more evidence is needed. In this regard, it is interesting to highlight that we identified four cases of overt Diabetes Mellitus (DM). These cases were presumably pre-pregnancy unnoticed cases of DM rather than frank GDM, but their identification underlines the interest of a simplified policy of glucose-intolerance screening. Indeed, if not identified, women with such severe glucose intolerance are exposed to life-threatening complications.

Our study also highlights some pitfalls of our intervention. First, the proportion of women who did not return for confirmation is too high. Reasons for drop-out were not actively investigated. However, we speculate that this may be mainly due to cultural reasons because the common local attitude is to overlook the importance of antenatal care. Most women in the studied settings refer only once during pregnancy. To note, the high proportion of nulliparous despite being in a high-fertility area also suggests that antenatal care was not perceived as crucial in the population (multiparae presumably skip any assessment during pregnancy). In addition, we cannot also rule out that logistic and financial constraints may have also played a role. This latter point is particularly concerning since the study refers to a period that was financially supported (tests and consultations were for free and women requiring confirmation were refunded for the costs of the journey) and the situation may worsen once external support will terminate. On the other hand, we are confident that the notion that fasting before performing an antenatal care consultation can progressively diffuse in the community so that, in the future, one may foresee to delete this step (or to reduce the proportion of women requiring to return). So far, our results show that confirmation is still a crucial aspect (only 22% of cases remains positive at confirmation) but also suggest that the parallel awareness campaign made during the project gave some good results given that confirmation was needed in a minority of women, specifically 27% (984 + 842/6641).

Second, the high rate of obstetrics complications in detected cases is troublesome. On the one hand, it confirms the idea that the strategy could have identified cases at worse prognosis, while on the other hand, it clearly underlines that clinical management has to be improved. Even if the diagnosis of GDM may be per se a therapeutic achievement because affected cases are aware of the increased risk of complications (obstructed labour, preeclampsia or neonatal maladaptation) and could refer earlier and to more equipped centers, efforts should be taken to improve prevention of complications with more effective life-habit consultations and with the increased use of the pharmacological armamentarium (metformin, glyburide or insulin). Referral for weekly monitoring was low and we did not attempt to actively investigate the adherence to diet and therapeutic prescriptions. The project did not foresee sufficient attention to this important but challenging part of the management of GDM. Planning specific training at the hospital and in the community and implementing a dedicated team for therapy could fill this gap.

Some limitations of the study deserve to be commented. First, inference made on the prevalence of GDM is theoretical and should be interpreted with caution. Even if we did not show significant differences in the available baseline characteristics between women who did and did not return for confirmation (data not shown), we cannot exclude some other relevant selection bias. Second, even if the study was implemented within an everyday clinical activity of antenatal care, there is a need for validation in other contexts. In particular, one has to investigate the acceptability and effectiveness in rural areas. Third, economical evaluations were not done. Implementation of any policy needs to be supported by in-depth and reliable cost–benefit analyses, in particular in remote settings. Unfortunately, our study was not designed to address this issue. We calculated that the crude costs for running the service (including consumable, salaries, refunds for confirmation-related journeys and costs of the glucometers) corresponded to 170 Euros per identified case of GDM (data not shown) but we were unable to quantify the benefits. Much larger samples with precise information on the outcomes are necessary. To note, given the difficulties in designing such studies, one may consider theoretical rather than real-life economic analyses.

## Conclusion

GDM is not rare in low-resource countries and may deserve consideration in public health interventions. Our protocol of diagnosis has some pros and cons but merits consideration, in particular if validated in other contexts. However, the strategy needs to be improved and several challenges remain. Priorities include the need to enhance sensitivity without increasing costs and the diffusion of the clinical know-how among healthcare givers.

## References

[1] UN. The Millennium Development Goals Report 2015. New York: United Nations 2015. https://www.un.org/millenniumgoals/2015_MDG_Report/pdf/MDG%202015%20rev%20(July%201).pdf. Accessed 09 July 2020

[2] Macaulay S, Dunger DB, Norris SA (2014). Gestational diabetes mellitus in Africa: a systematic review. PLoS ONE.

[3] Behboudi-Gandevani S, Amiri M, Bidhendi Yarandi R, Ramezani Tehrani F (2019). The impact of diagnostic criteria for gestational diabetes on its prevalence: a systematic review and meta-analysis. Diabetol Metab Syndr.

[4] Mack LR, Tomich PG (2017). Gestational diabetes: diagnosis, classification, and clinical care. Obstet Gynecol Clin North Am.

[5] Lowe WL, Scholtens DM, Lowe LP (2018). HAPO follow-up study cooperative research group. Association of gestational diabetes with maternal disorders of glucose metabolism and childhood adiposity. JAMA.

[6] Johns EC, Denison FC, Norman JE, Reynolds RM (2018). Gestational diabetes mellitus: mechanisms, treatment, and complications. Trends Endocrinol Metab.

[7] Hod M, Kapur A, McIntyre HD, FIGO working group on hyperglycemia in pregnancy and the FIGO pregnancy and prevention of early NCD committee (2019). Evidence in support of the international association of diabetes in pregnancy study groups’ criteria for diagnosing gestational diabetes mellitus worldwide in 2019. Am J Obstet Gynecol.

[8] HAPO study cooperative research group (2008). Hyperglycemia and adverse pregnancy outcomes. N Engl J Med.

[9] Crowther CA, Hiller JE, Moss JR, McPhee AJ, Jeffries WS, Robinson JS, Australian carbohydrate intolerance study in pregnant women (ACHOIS) trial group (2005). Effect of treatment of gestational diabetes mellitus on pregnancy outcomes. N Engl J Med.

[10] Landon MB, Spong CY, Thom E (2009). A multicenter, randomized trial of treatment for mild gestational diabetes. N Engl J Med.

[11] Hod M, Pretty M, Mahmood T, FIGO, EAPM and EBCOG (2018). Joint position statement on universal screening for GDM in Europe by FIGO, EBCOG and EAPM. Eur J Obstet Gynecol Reprod Biol.

[12] Coustan DR, Lowe LP, Metzger BE, Dyer AR, International association of diabetes and pregnancy study groups (2010). The hyperglycemia and adverse pregnancy outcome (HAPO) study: paving the way for new diagnostic criteria for gestational diabetes mellitus. Am J Obstet Gynecol.

[13] Nielsen KK, de Courten M, Kapur A (2012). Health system and societal barriers for gestational diabetes mellitus (GDM) services: lessons from world diabetes foundation supported GDM projects. BMC Int Health Hum Rights.

[14] Nielsen KK, Kapur A, Damm P, de Courten M, Bygbjerg IC (2014). From screening to postpartum follow-up - the determinants and barriers for gestational diabetes mellitus (GDM) services, a systematic review. BMC Pregnancy Childbirth.

[15] UN, 2018. United Nations development programme human development reports https://hdr.undp.org/en/2018-update Accessed April 6th, 2019

[16] WHO, 2015. Global Health Observatory country views https://apps.who.int/gho/data/node.country.country-SLE Accessed April 6th, 2019

[17] UNICEF United Nation Children Funds. Monitoring the situation of children and women. https://data.unicef.org/country/sle/ (Accessed April 6th, 2019)

[18] Sierra Leone 2015 Population and housing census. Thematic report on population projections https://www.statistics.sl/images/StatisticsSL/Documents/Census/2015/sl_2015_phc_thematic_report_on_population_projections.pdf. Accessed 09 July 2020

[19] Catalano PM (2014). Trying to understand gestational diabetes. Diabet Med.

[20] Seyoum B, Kiros K, Haileselase T, Leole A (1999). Prevalence of gestational diabetes mellitus in rural pregnant mothers in northern Ethiopia. Diabetes Res Clin Pract.

[21] Adam S, Rheeder P (2017). Screening for gestational diabetes mellitus in a South African population: prevalence, comparison of diagnostic criteria and the role of risk factors. S Afr Med J.

[22] Nielsen KK, de Courten M, Kapur A (2012). The urgent need for universally applicable simple screening procedures and diagnostic criteria for gestational diabetes mellitus–lessons from projects funded by the World Diabetes Foundation. Glob Health Action.

[23] Hod M, Kapur A, Sacks DA (2015). The International Federation of Gynecology and Obstetrics (FIGO) Initiative on gestational diabetes mellitus: a pragmatic guide for diagnosis, management, and care. Int J Gynaecol Obstet.

[24] Adam S, Rheeder P (2018). Evaluating the utility of a point-of-care glucometer for the diagnosis of gestational diabetes. Int J Gynaecol Obstet.

